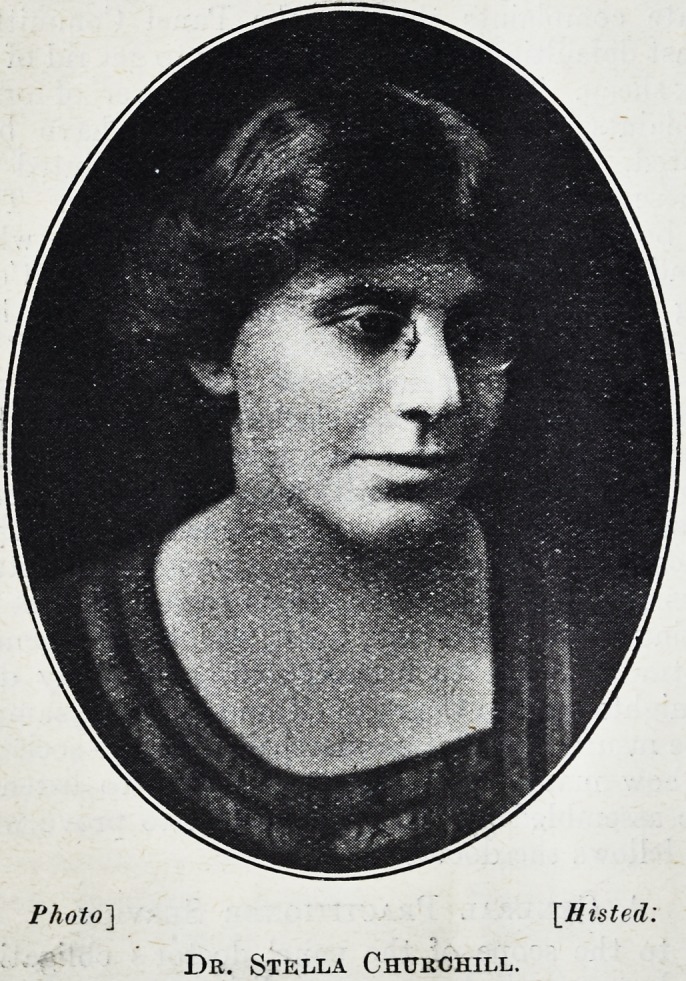# Health Weeks in London

**Published:** 1924-01

**Authors:** Stella Churchill


### HEALTH WEEKS IN LONDON.

### THEIR USEFULNESS AND PROSPECTS.

**By STELLA CHURCHILL, M.R.C.S., L.R.C.P., D.P.H.**

THE movement known as Health Week was started in 1912 to focus attention for at least one week in the year on the subject of health. The war interrupted the development of this idea, but for the last five years Health Week has been celebrated throughout the country in a variety of ways. The fundamental idea underlying the scheme is to arouse a sense of personal responsibility for keeping well. Too long has the medical profession devoted its energies entirely to the curing of

*Photo*] [*Histed*.

Dr. Stella Churchill.

the sick; now the advent of preventive medicine augurs, let us hope, a new era for mankind. The idea that disease can be arrested at its beginning, and much misery and sorrow saved, has received scientific as well as popular encouragement; and it is from the popular side—to bring the matter home to the man in the street—that Health Week was originally founded. A certain amount of apathy exists over this question of health, and there are only too many people who "enjoy" bad health to the detriment of themselves and their neighbours. It is sometimes suggested that to focus attention on health may awaken an undesirable curiosity as to the symptoms of disease, but the object of preventive medicine must, to some extent, be to arouse such a curiosity so that we may no longer have the spectacle of patients in advanced stages of phthisis, cancer, syphilis, heart trouble, etc., coming to hospital for the first time. To create a public opinion which shall demand health as one of its rights is the way to arrest disease at its earliest stage, or, better still, to prevent its onset:—

> Ignorance is the curse of God,
> Knowledge the wing wherewith we fly to heaven.

### Authorities Concerned.

The development of Health Week in London has been left by the founders to the initiative of each individual borough. The aim is to educate all ages of the community in this preventive campaign, beginning with the mother and the new-born infant, and following the child through the successive stages of youth and adolescence up to adult life. In London, at any rate, a difficulty arises in the failure to secure that co-operation which should exist between all branches of public health services, because the schools are under the authority of the London County Council and are in no way controlled by the Medical Officer of Health. Some boroughs, however, have found it practical to have lectures specially arranged for school children, which lectures are allowed by the Board of Education to count as part of the school curriculum. The education of the parents is also to some extent achieved through the agency of the school. Where two authorities are concerned in arranging propaganda it is desirable to avoid overlapping in drawing up the programme. The sympathy and co-operation of all classes of the community must be secured on the preliminary committee, and the teachers are enthusiastic, as a rule, in their support.

### The Demand for Knowledge.

Practical experience of Health Week in a London borough has taught me that there is a very growing demand on the part of the public for knowledge on the subject of "how to keep well." More particularly do they want information on the problems of everyday life, on the choice and preparation of foods, on the kind of clothing for their children and the number of garments that need be worn, on the need for fresh air and how to secure ventilation without a "draught," on the normal functions of the body and their daily use without the abuse of drugs. These and a dozen other cognate questions may form subjects for debate.

### Co-operation of the Public Health Staff.

All members of a public health staff can assist in the conduct of Health Week, not only the Medical Officer of Health, who must organise and arrange a scheme, but also the sanitary and food inspectors, who can give valuable technical information at short lectures and demonstrations on food values, drainage, house construction, etc. Infant Welfare Centres can make a special point of lectures and health talks to mothers and co-operate by personally conducting parties of mothers to public lectures given at the town hall or some other central building. It should be possible to enlist the sympathy and support of all large employers of labour. To put it at its very lowest, one can appeal to the self-interest of the business man by pointing out that health goes hand in hand with efficiency. In some instances short mid-day talks to factory hands have been successfully arranged with the co-operation of the head of the firm. Lecturers can usually be found who will give such talks on venereal diseases, or tuberculosis, or other subjects that may be suitable to the environment.

### Value for Money.

Health Week gives a unique opportunity of pointing out to the general public what good value they get for the rates. The sanitary condition of a city like London compares favourably with that of any of the large continental cities which approach it in size. We are inclined to take this freedom from offensive smells and other nuisances too much for granted. Municipal authorities are as a rule quite ready and proud to have a public demonstration of their efficiency in the shape of their dust destructor, disinfecting station and other public works. The sympathy of the Mayor will be elicited in all branches of the work, and it is common experience that all shades of political opinion will combine to further the cause of health in the borough.

### What Propaganda Will Do.

Health Week, in short, is one of the best ways in which we can develop and foster a true spirit of citizenship. It involves consideration for others just as much as for ourselves. Public opinion can, and will, no doubt create a very stirring demand for proper housing, better laws in respect of the sale and preparation of food, and a higher standard in the personal hygiene of daily life. If the propaganda work carried out during Health Week arouses in only a very small proportion of those who attend the lectures and demonstrations a demand for better conditions it will not have been without great success, and every child brought up and educated in this spirit will be enabled to make fuller use of its life. Finally, the ever-increasing burden of avoidable sickness and ill-health, which to-day overcrowds the in-patient and out-patient departments of our hospitals, will inevitably diminish.

## Figures and Tables

**Figure f1:**